# Wavelength-dispersive spectrometer for X-ray microfluorescence analysis at the X-ray microscopy beamline ID21 (ESRF)

**DOI:** 10.1107/S0909049510010691

**Published:** 2010-04-01

**Authors:** J. Szlachetko, M. Cotte, J. Morse, M. Salomé, P. Jagodzinski, J.-Cl. Dousse, J. Hoszowska, Y. Kayser, J. Susini

**Affiliations:** a European Synchrotron Radiation Facility, Grenoble, France; b Institute of Physics, Jan Kochanowski University, Kielce, Poland; c Centre of Research and Restoration of French Museums, UMR171, Paris, France; d Technology University, Kielce, Poland; e Department of Physics, University of Fribourg, Fribourg, Switzerland

**Keywords:** X-ray spectroscopy, wavelength-dispersive spectrometer, X-ray imaging

## Abstract

The development of a wavelength-dispersive spectrometer for microfluorescence analysis at the X-ray Microscopy ID21 beamline of the European Synchrotron Radiation Facility (ESRF) is reported. The spectrometer is based on a polycapillary optic for X-ray fluorescence collection and is operated in a flat-crystal geometry. The design considerations as well as operation characteristics of the spectrometer are presented. The achieved performances, in particular the energy resolution, are compared with the results of Monte Carlo simulations. Further improvement in the energy resolution, down to ∼eV range, by employing a double-crystal geometry is examined. Finally, examples of applications requiring both spatial and spectral resolutions are presented.

## Abstract 2

A polycapillary-based wavelength-dispersive spectrometer is reported. The design consideration as well as operation characteristics are presented.

## 1. Introduction

The ESRF ID21 beamline is dedicated to X-ray microscopy and microanalysis. It relies on a submicrometre X-ray probe in the energy range between 2 keV and 7.2 keV and is mainly oriented to micro-X-ray fluorescence and micro-XANES (X-ray absorption near-edge structure) analysis. Owing to its unique combination of high spatial resolution and spectroscopy capability with a low detection limit, the beamline attracts a broad user community, covering environmental and materials science, medicine, biology and archeometry. It is worthy to note that there is an increasing demand for the development of new and complementary X-ray techniques based on high spatial resolution and high-sensitivity X-ray fluorescence detection. Until now, the ID21 beamline relied on several solid-state detectors, which are complementary in terms of count-rate throughput and solid-angle collection efficiency. However, the attainable energy resolution (120–180 eV) of such energy-dispersive detectors is often inadequate to permit unequivocal elemental and chemical speciation. To improve the energy resolution of fluorescence detection of the X-ray microscope, a wavelength-dispersive spectrometer has been developed for the specific requirements of the ID21 beamline.

Since 1913, when the Bragg law was formulated (Bragg & Bragg, 1913 ▶), wavelength-dispersive spectroscopy has become one of the most important tools in X-ray fluorescence analysis. In contrast with energy-dispersive detectors, wavelength-dispersive spectrometers (WDSs) can provide a much better energy resolution of the detected X-rays. Wavelength-dispersive spectroscopy is now a well established technique at synchrotron facilities. It is widely used in both X-ray fluorescence and X-ray absorption analysis. The combination of monochromatic beam and high-resolution spectroscopy also make possible studies of the X-ray resonant Raman scattering (Kotani & Shin, 2001 ▶).

**Figure 1:**
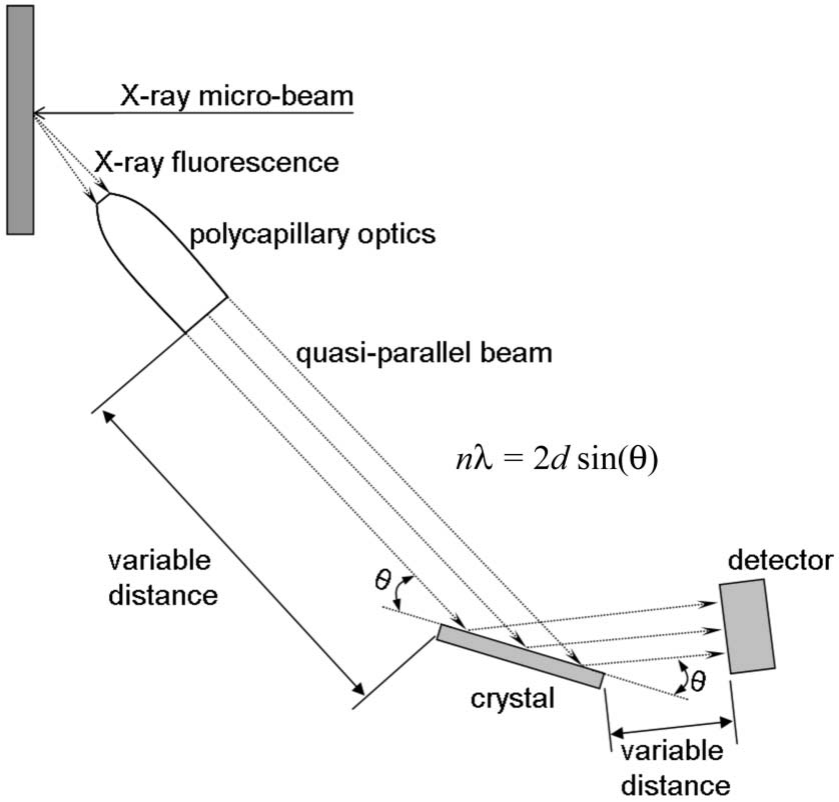
Schematic drawing of the parallel-beam wavelength-dispersive arrangement employing polycapillary optics for X-ray fluorescence collection.

The arrangement of a WDS is usually based on the curved crystal geometry (Johann, 1931 ▶; von Hamos, 1932 ▶; Johansson, 1933 ▶; DuMond, 1947 ▶). This enhances the efficiency of the instrument, but results in demanding and complicated alignment and operation. Moreover, such instruments require a lot of space for installation because of the Rowland circle geometry. In consequence, they are not practical for the existing experimental set-up of the beamline ID21. Recently, a number of parallel-beam wavelength-dispersive spectrometers (PB-WDSs) have been developed (Soeima & Narusawa, 2001 ▶; Schields *et al.*, 2002 ▶; Hoek & Koolwijk, 2008 ▶; Parallax, http://www.parallaxray.com/wds.html) which employ polycapillary optics for the X-ray fluorescence collection and flat crystals for the X-ray diffraction. By the nature of their design, these PB-WDSs are well suited to X-ray fluorescence analysis using electron or photon primary beams that have been focused down to the micrometre scale. However, to our knowledge, until now the PB-WDS arrangement has not been reported for synchrotron micro-beam excitations. Substantial progress has also been made during recent years in the performance of polycapillary optics, and, in consequence, the performance of recent PB-WDSs can be much improved over previous systems. The schematic geometry of the PB-WDS configuration is shown in Fig. 1 ▶. The polycapillary optics is placed at a distance of a few millimetres from the sample. This optics converts the divergent X-ray fluorescence emitted from the sample into a quasi-parallel beam, which is then directed onto the flat crystal at the required Bragg θ angle. The X-rays diffracted by the crystal are counted by a detector placed at the 2θ angle. The PB-WDS configuration results in a simple alignment of both crystal and detector. Moreover, the polycapillary-to-crystal and crystal-to-detector distances are not critical and thus can be easily adapted to specific experimental constraints. This arrangement also permits a very compact geometry, which represented a necessary prerequisite for the installation of a high-resolution X-ray microfluorescence analysis system at the ID21 beamline.

## 2. Spectrometer design

### 2.1. General description of the ID21 beamline

**Figure 2:**
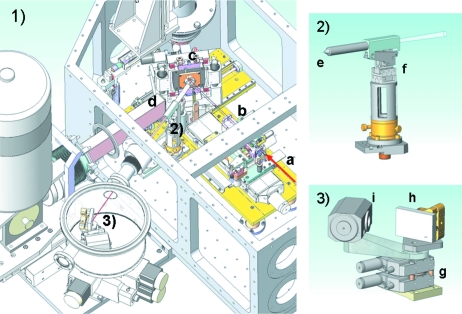
Technical drawing of (1) the scanning X-ray microscope of ID21 (for clarity, only the main elements are shown), (2) the polycapillary stage and (3) the θ–2θ stage. Labels: *a*, primary X-ray beam; *b*, focusing optics table; *c*, sample stage; *d*, seven-element HpGe detector; *e*, PB-WDS polycapillary optics; *f*, *xyz* piezo stage; *g*, θ–2θ rotation; *h*, crystal; *i*, gas-flow detector.

The ID21 X-ray microscopy beamline is designed for a spectral range between 1.0 keV and 7.2 keV. For X-rays above 2 keV the white beam is delivered by U40 and/or U42 undulators, whereas for lower energies a wiggler W80 is employed. The higher-order harmonics are rejected by a fixed-exit two-bounce mirror system working at a glancing angle of a few milliradians. Depending on the beam energy, Si, Rh or Ni mirror coatings can be chosen. The double-mirror system allows for harmonics rejection at the level of 10^−4^ with a transmission higher than 70%. The X-rays are monochromated by a fixed-exit double-crystal monochromator (Kohzu, Japan) employing either Si(111) or Si(220) crystals, or a Ni/B_4_C multilayer mirror. The monochromatic X-ray beam then passes into the scanning X-ray microscope (SXM), which is schematically shown in Fig. 2(1) ▶. In the SXM, the X-ray beam can be focused by means of Fresnel zone plates or a Kirkpatrick–Baez mirror arrangement placed on the optics stage. A beam size of 0.3 µm × 0.7 µm is commonly achieved with a photon flux of 10^9^–10^10^ photons s^−1^ depending on the beam energy. The sample is raster scanned across the beam focal point to build up a two-dimensional elemental or chemical image. The X-ray fluorescence signal is recorded by a seven-element HpGe (Princeton Gamma-Tech, USA) or a single-element silicon drift diode (SDD) detector (Bruker AXS, Germany), placed at 90° or 60° scattering angles, respectively. In the case of sample transmission or phase-contrast measurements, a Si diode placed downstream of the sample is used to record variations in the transmitted beam intensity. Owing to the wide range of available beam energies and detection modes, the ID21 SXM allows for detection and chemical speciation of almost all elements of the periodic table (Susini *et al.*, 2002 ▶). The microscope can be operated either at atmospheric pressure or under vacuum (10^−6^ mbar).

### 2.2. The parallel-beam wavelength-dispersive spectrometer

The PB-WDS was designed in a way to be fully integrated within the SXM of the ID21 beamline. However, owing to space constraints and practical issues of operating the existing SXM, the PB-WDS was divided into two main parts: the polycapillary stage [Fig. 2(2) ▶] and the θ–2θ stage [Fig. 2(3) ▶]. The polycapillary stage is installed inside the SXM at a distance of a few centimetres from the sample, while the θ–2θ stage is mounted within a separate chamber that is itself mounted to the SXM using a 60° vacuum port originally reserved for the SDD detector. The distances between the polycapillary stage and θ–2θ stage and between the crystal and detector are about 60 cm and 7 cm, respectively. The design allows for simple and rapid exchange of the SDD detector and the PB-WDS depending on the needs of a particular experiment.

### 2.3. Polycapillary optics stage

The polycapillary optics stage [Fig. 2(2) ▶] enables precise alignment of the polycapillary optics with respect to the X-ray beam focal spot on the sample: it uses an *xyz* piezo linear stage (Mechonics MS-30, http://www.mechonics.de/). The resolution of the three linear movements is about <0.5 µm with a travel range of 8 mm for each axis. For the adjustment of the horizontal and vertical angles, two manual rotations are integrated in the device.

The main specifications of the polycapillary optics, manufactured by XOS (http://www.xos.com/), are quoted in Table 1 ▶. The parameters of the optics were optimized taking into account the specific X-ray beam parameters of ID21, in particular the beam focus size on the sample and the required X-ray fluorescence detection energy range.

### 2.4. θ–2θ stage and crystals

The θ–2θ stage, shown in Fig. 2(3) ▶, consists of two rotation motors, the crystal and the detector. The rotation stage (Micos RS-40, http://www.micos.ws/index.html) enables angle adjustment with a precision of 3 mdeg. The two motors are mounted together to form a compact goniometer stage and to ensure the co-centricity of the two rotation axes. The θ–2θ rotation allows for Bragg angles between 20° and 70°.

Two different flat crystals are used for X-ray diffraction: Ge(220) (2*d* = 4.000 Å) and Si(111) (2*d* = 6.271 Å). Both crystals are 6 cm wide and 4 cm high. The optical holder allows for the vertical and horizontal alignments of the crystal axes by means of three adjustment screws placed behind the crystal.

### 2.5. Detection system

For X-ray detection, a gas-flow proportional counter (Parallax, http://www.parallaxray.com/wds.html) is employed. This detector has an ultra-thin polymer window 22 mm in diameter (http://www.moxtek.com/X-ray/proportional.html), and is operated with an anode wire voltage of 1.7–1.9 kV. A standard P10 gas counter mixture (10% methane and 90% argon) is flowed through the detector at atmospheric pressure at a rate of about 25 s.c.c.m. The detector anode signal is amplified with an eV5093 preamplifier (eV Products, 2009 ▶) and the signal processed using the same XIA DXP-XMAP electronics and software (http://www.xia.com/) as are used for the SXM energy-dispersive detectors. The XMAP provides online control of the energy window discrimination used for the signal pulses from the proportional counter.

## 3. Experimental results

### 3.1. Spectrometer adjustments/alignment

A series of X-ray fluorescence spectra between 2 keV and 7.2 keV were measured. Metal foils or reference powders were used as standards. The monochromatic X-ray beam was focused to a size of 0.6 µm × 1 µm with a photon flux of about 10^9^ –10^10^ photons s^−1^. The polycapillary, pre-adjusted using a laser system, was aligned to the beam spot on the sample by scanning it in the vertical and horizontal directions and recording the corresponding X-ray signal intensity with the gas detector. For this, the detector was placed at a 0° θ angle (*i.e.* parallel to the X-ray polycapillary axis) with the crystal dismounted from the θ–2θ stage. The focal distance, *i.e.* the distance between the sample and entrance of the polycapillary, was adjusted in a similar way. For the energy calibration of the spectrometer, θ–2θ scans of reference X-ray fluorescence lines were performed.

**Figure 3:**
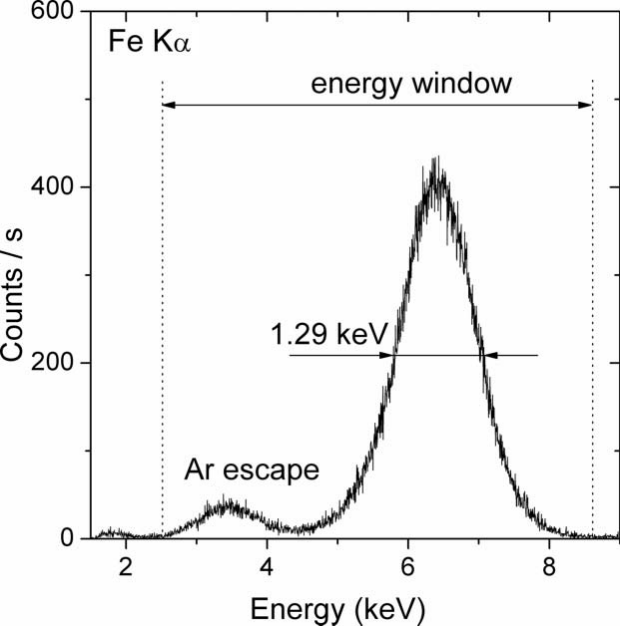
Energy spectrum recorded with the Parallax gas proportional counter detector for the Fe *K*α fluorescence line.

In order to remove the higher orders of reflection from the crystal and spurious low-energy events from the gas detector noise, only the X-ray events within a suitable energy window are counted. The appropriate energy window is determined based on the energy spectrum recorded by the gas detector at the Bragg angle corresponding to the energy of the X-ray fluorescence line of interest. As an example, the *K*α line of the energy spectrum measured with a Fe foil is shown in Fig. 3 ▶. The spectrum consists of the main peak centred at 6.4 keV corresponding to the Fe *K*α line and a peak around 3.6 keV. The latter, which has an intensity of about 6% of the Fe photopeak, corresponds to Ar *K* X-rays escaping from the detector. The energy resolution obtained with the gas detector is about 20% (FWHM). To count the photons, an energy window covering both peaks was chosen, as indicated in Fig. 3 ▶.

The PB-WDS X-ray fluorescence spectrum from the sample is recorded by a point-by-point scan through the corresponding Bragg angle domain. Depending on the measured energy, steps of 1 eV to 5 eV are used with a typical exposure time of 1 s to 5 s per point. The full X-ray fluorescence spectrum (*i.e.* containing all the main X-ray transitions) consists of 300–500 points. Therefore the total acquisition time needed for data collection is about 5–10 min per spectrum. For each measured point, only the photons that fall into the energy window are integrated, and when the measured X-ray energy is changed by more than ∼500 eV the energy discrimination window is redefined. Similarly, the polycapillary focal position is adjusted and optimized for each new spectrum in order to maintain a maximum X-ray throughput.

### 3.2. X-ray fluorescence spectra and detection limits

**Figure 4:**
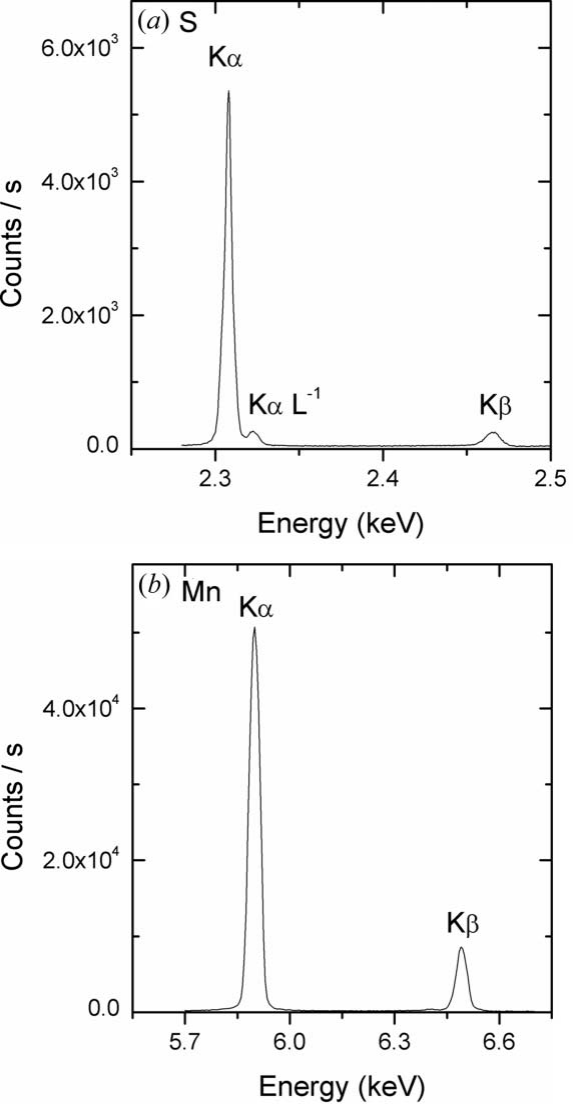
Examples of X-ray fluorescence spectra recorded with the spectrometer: (*a*) CaSO_4_.2H_2_O powder, recorded with a Si(111) crystal, (*b*) Mn foil, recorded with a Ge(220) crystal.

As an example, the X-ray fluorescence spectra of sulfur from a CaSO_4_.2H_2_O powder sample and manganese from a Mn metal foil are shown in Figs. 4(*a*) and 4(*b*) ▶, respectively. Clearly, the resolution of the spectrometer gives a very good separation of the *K*α and *K*β emission lines. In the case of sulfur, a small peak on the high-energy side of the *K*α fluorescence can be observed that corresponds to the *K*α*L* ^−1^ satellite line produced by the shake process (Mauron *et al.*, 2000 ▶). Owing to their relatively small energy difference (*i.e.* ∼20 eV) with respect to the main emission line, these satellite transitions are not resolved when using a solid-state detector.

High X-ray fluorescence count rates, at the level of 10^4^–10^6^ counts s^−1^ in the peak intensity, can be obtained owing to the large solid angle covered by the polycapillary. The detection limits (DLs) for the present PB-WDS set-up were determined for several elements using NIST-610 and -612 glass-matrix standard samples (NIST, https://www-s.nist.gov/srmors/view_detail.cfm?srm=613). DLs of 35 p.p.m. for Fe (*K*α = 6.404 keV, *E*_beam_ = 7.2 keV), 23 p.p.m. for Ti (*K*α = 4.511 keV, *E*_beam_ = 5.5 keV) and 46 p.p.m. for K (*K*α = 3.314 keV, *E*_beam_ = 4.6 keV) were obtained, all at 1 s exposure time and with incident photon flux in the range 2 × 10^10^ to 5 × 10^10^ photons s^−1^. However, the DLs can change depending on the particular set-up of the ID21 beamline. For example, employing the Ni/B_4_C multilayer monochromator leads to a more intense beam (∼5 × 10^11^ photons s^−1^) with a slightly worse lateral beam resolution (a few µm). For such a set-up the DL of 5.6 p.p.m. for K was achieved. The DLs could also be further improved by employing a dedicated shielding around the detector window in order to prevent the detection of X-rays scattered around the θ–2θ stage. In addition, a significant enhancement of the DLs is expected if a multilayer analyzer (instead of a crystal) is used for the X-ray diffraction. These technical improvements will be tested in the future.

### 3.3. Spectrometer resolution

The instrument response and energy resolution for the PB-WDS geometry depend primarily on the beam divergence at the output of the polycapillary. The polycapillary optics collects those X-rays emitted from the sample which meet its critical angle criterion, and these photons are transmitted through the polycapillary by multiple internal reflections. The output X-ray beam therefore comprises many photon energies which are divergent at different angles with respect to the optics output axis. When this divergent beam is directed upon the flat crystal, only those photons that fulfil the Bragg criteria are diffracted. However, this occurs for slightly different photon energies depending on the direction of propagation of each photon, and as discussed below; in practice, this determines the spectrometer resolution.

**Figure 5:**
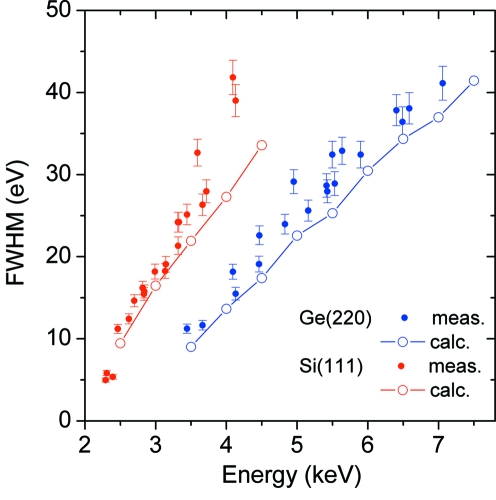
Measured energy resolution (full circles) of the spectrometer equipped with a Si(111) (in red) and Ge(220) (in blue) crystal. Results of the Monte Carlo simulations are depicted by open circles.

The experimental spectrometer resolution, which was found to be well reproduced by a Gaussian function, was determined by a fitting procedure applied to each measured spectrum. The results obtained as a function of the measured photon energies are presented in Fig. 5 ▶ for both Si(111) and Ge(220) crystals. For the Ge crystal, a resolution between 40 eV at 7.1 keV and 10 eV at 3.5 keV was achieved. Similar results were obtained for the Si crystal, with values between 35 eV at 4.5 keV and 5 eV at 2.3 keV.

The theoretical dependence of the energy resolution as a function of the measured X-ray energy can be explained as follows. The energy resolution Δ*E* of the spectrometer is deduced *via* Bragg’s law according to
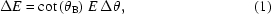
where *E* is the energy and θ_B_ is the Bragg angle of the measured X-ray fluorescence line. In the case of the PB-WDS, Δθ represents the convolution of the beam divergence θ_Beam_ with the rocking curve θ_Crystal_ of the crystal. However, in this case θ_Crystal_ is an order of magnitude smaller than the beam divergence and so can be neglected. The experimental results show that for the energy range between 3.4 keV and 4.2 keV the energy resolution of the spectrometer with the Ge(220) crystal was about two times better than for the Si(111). Since θ_Beam_ was the same in both cases (*i.e.* measurements were made at the same energy), the difference in resolution must have resulted from the different Bragg angles characterizing the two measurements. In the case of Ge(220), the Bragg angles in the energy range 3.4–4.2 keV are large (50–65°) and therefore the factor cot(θ_B_), which describes the energy resolution in (1), is small. The opposite is observed with the Si(111) crystal since the Bragg angles for the same energy range are between 28 and 35° and therefore the factor cot(θ_B_) is correspondingly larger. These results demonstrate that the energy resolution of the instrument can be optimized by employing suitable crystals for the X-ray diffraction, and that the best energy resolution is obtained when the spectrometer is operated at relatively large Bragg angles.

To calculate the energy resolution as a function of the measured X-ray energy, the beam divergence at the output of the polycapillary needs to be accurately known. This parameter, which is proportional to the critical angle, depends on many other factors like the spot size of the primary X-ray beam, the input focal distance and the dimensions and shape of the polycapillary. For this reason, Monte Carlo simulations have been performed for X-ray tracing in the PB-WDS geometry. In our computations, each capillary was assumed to have a cylindrical shape, the cylinder axis being bent according to the function *y* = *C*_1_ *x* ^3^ + *C*_2_ *x* + *C*_3_ with the boundary condition *y*′(*L*) = 0, where *L* is the length of the capillary and *C*_1_, *C*_2_ and *C*_3_ are geometrical factors that depend on its length, size and bending radius. Note that the bending radius of a capillary changes with its radial distance. A diameter of 10 µm was taken for the capillaries at the entrance side, whereas their diameter at the exit side was multiplied by the ratio of the exit-to-entrance radius of the entire polycapillary bundle. X-ray spectra were calculated assuming a point-like source on the sample with isotropic emission of X-rays. For each energy, 10^6^ photons were generated with random directions of propagation. The internal total-reflection probabilities in the capillaries were calculated using the scattering factors tabulated by Henke *et al.* (1993 ▶), and the diffraction by the crystal was simulated employing the rocking curves provided by the *XOP* program (http://www.esrf.eu/computing/scientific/xop2.0/download.html). The calculated energy resolutions for the Si(111) and Ge(220) crystals are presented in Fig. 5 ▶. As can be seen, the results of Monte Carlo simulations are in reasonable agreement with the experimental data, but the measured data points all lie slightly above those calculated. This is most likely due to the differences between the real and simulated polycapillary performances. However, the agreement between the experimental data and the Monte Carlo calculations is sufficiently good to use the simulations as a tool to optimize the spectrometer design when extending its spectral range to lower energies.

### 3.4. Future improvement of the spectrometer resolution

As discussed, the energy resolution of the PB-WDS depends mainly on the divergence of the X-ray beam delivered by the polycapillary optics. Therefore, to further improve the instrumental resolution, the beam divergence must be reduced. To this end, Soller slits placed between the polycapillary and the diffracting crystal could be used. However, a drawback of this method is that a decrease in the beam divergence will be accompanied by substantial losses in the transmitted X-ray intensities. We show that a more efficient solution can be obtained based on a double-crystal geometry.

**Figure 6:**
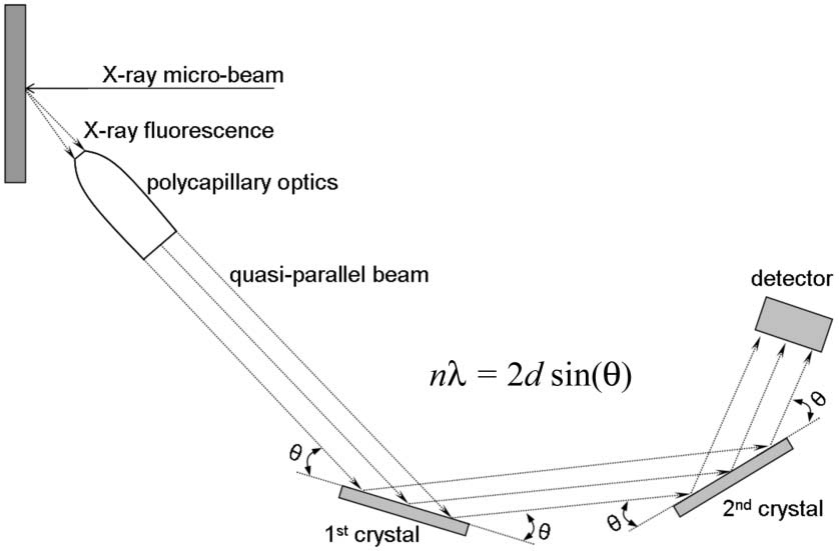
Schematic drawing of the PB-WDS employing two plane crystal analyzers.

The double-crystal arrangement for the PB-WDS operated in the anti-parallel geometry is schematically shown in Fig. 6 ▶. Here, it is important to note that after diffraction by the first crystal the X-ray beam becomes ordered in space, *i.e.* the direction of propagation of the diffracted photons depends strictly on their energy. Only those photons that fulfil the Bragg criteria will undergo diffraction by the second crystal. The double-crystal set-up enables a reduction in the beam divergence down to about microradian levels, so that the energy resolution of the spectrometer will depend only on the Darwin width of the crystals which is of the order of 10^−4^ rad. To investigate the performance of the double-crystal set-up a preliminary test was performed using two Ge(220) crystals. Only a slight modification of the initial PB-WDS set-up was needed, involving the mounting of the second crystal on the detector 2θ arm. Since the detector was mounted on the same arm, the X-rays entered the detector window at the θ angle. This simple double-crystal set-up allowed us to perform test measurements for Bragg reflections between 25° and 35°.

**Figure 7:**
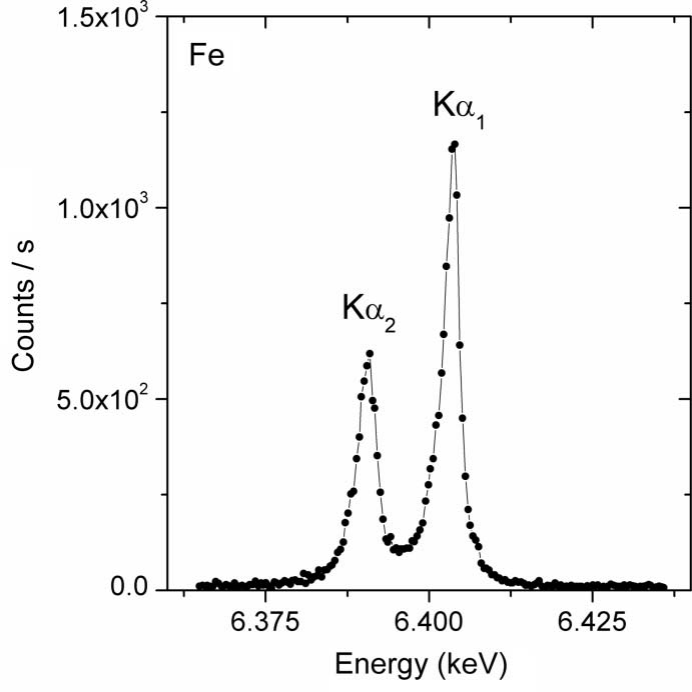
The Fe *K*α fluorescence measured with the PB-WDS operated in the double-crystal geometry.

The Fe *K*α fluorescence measured with the double-crystal PB-WDS is shown in Fig. 7 ▶. The resolution achieved is more than sufficient to resolve the *K*α_1,2_ doublet resulting from the spin–orbit interaction. The measured peak widths were 2.77 eV and 3.70 eV for the *K*α_1_ and *K*α_2_ emission lines, respectively. Taking into account the natural line widths of these transitions which are 1.6 eV for *K*α_1_ (*K*-*L*_3_) and 2.33 eV for *K*α_2_ (*K*-*L*_2_), we deduced the resolution of the spectrometer itself to be about 2.0 eV. Thus, the relative resolution Δ*E*/*E* for the double-crystal geometry was determined to be 3.1 × 10^−4^. The measured photon count rates were reduced by a factor of ∼20 as compared with the single-crystal geometry.

The results obtained show that the double-crystal PB-WDS arrangement presented here can be an alternative to other wavelength-dispersive geometries. Its overall size does not greatly exceed that of the compact optical elements it uses, and the polycapillary-to-crystal, crystal-to-crystal and crystal-to-detector distances are not crucial. High resolution is achieved without the constraints imposed by the Rowland circle geometry. For the present set-up the total X-ray path amounts to only 15 cm (3 cm polycapillary length + 2 × 6 cm length crystals). The latter could be diminished yet further if needed by placing the crystals just behind the polycapillary optics, so that the entire system could in principle be restricted within an 8 cm space. This is an extremely attractive feature, as state-of-the-art experimental set-ups require the simultaneous use of several instruments, all of which compete for space within the immediate sample environment.

The double-crystal arrangement will be further developed to extend its energy range by employing other crystals. In addition, we will investigate the possibility of using a multilayer as the first X-ray diffractor and a crystal as the second one. This should enable a significant increase in the overall transmission efficiency of the double-crystal arrangement. The experimental energy resolution already achieved is comparable with that expected from the lifetimes of atomic core-holes: this allows chemical speciation at submicrometre lateral resolutions to be performed using the resonant inelastic X-ray scattering method. The latter has proved to be a unique technique which is complementary to XANES measurements (Kotani & Shin, 2001 ▶).

## 4. Examples of applications for micro-XANES and two-dimensional chemical mapping

As art objects often have complex and heterogeneous compositions, cultural heritage is one of the fields in which the PB-WDS will find many applications. Ancient paintings are the archetype of puzzling materials: they are often a complex mixture of tens of ingredients, inorganic and crystallized products such as pigments and plasters; organic and amorphous ingredients, such as binders, sizers or varnishes. The analysis of such complex materials requires measurements of high spatial resolution, owing to their micrometric heterogeneity, as well as high spectral energy resolution, owing to their chemical complexity (Cotte *et al.*, 2006 ▶, 2008 ▶). In particular, these samples often contain mixtures of high-*Z* elements (such as lead or mercury) together with low-*Z* elements. Overlap of *M*- or *L*-emission lines of the former with the *K*-emission lines of the latter hamper elemental identification with fluorescence analyses, and, here, high spectral resolution is essential.

X-ray absorption spectroscopy is also a powerful method for accessing element oxidation states and more generally their chemical environment within the museum artifacts. It is a unique tool which provides information about both the technologies used to manufacture the artwork and the reactions which alter and damage them (Cotte *et al.*, 2010 ▶). As an example, the determination of the oxidation state of metal transitions in ancient glasses can provide information about lost manufacturing technologies (choice of ingredients, firing temperature and atmosphere). Such an analysis has been recently carried out to highlight antimony-based opacification processes used in ancient Egypt (Lahlil *et al.*, 2010 ▶). The family of lead-oxides-based yellow pigments is a particularly interesting case. Such compounds were first used as glass colourants and opacifiers by Egyptians around 1600–1400 BC and were later introduced as pigments in easel paintings (Wainwright *et al.*, 1986 ▶). Depending on the time and place of production, they are made of lead and tin [with two distinct classes: lead tin yellow type I (Pb_2_SnO_4_) and lead tin yellow type II, formulated as PbSnO_3_ by Rooksby (quoted by Hradil *et al.*, 2007 ▶) or PbSn_*x*_Si_1–*x*_O_3_ by Clark *et al.* (quoted by Hradil *et al.*, 2007 ▶)] or lead and antimony [usually called Naples yellow (Pb_2_Sb_2_O_7_)]. A ternary Pb–Sb–Sn oxide has also been employed (Hradil *et al.*, 2007 ▶). In this context a strict elemental analysis is a first step to identify and differentiate these pigments. However, as demonstrated by Hradil *et al.* (2007 ▶), this analysis is not necessarily adequate as mixtures of several pigments can lead to a misinterpretation of the elemental composition. Characterizing the chemical formulation of the materials is more appropriate for pigment identification, where the objective is to establish the historical and geographical evolutions in the manufacturing processes and use of the chemicals employed. Raman and X-ray diffraction can also provide such characterizations.

**Figure 8:**
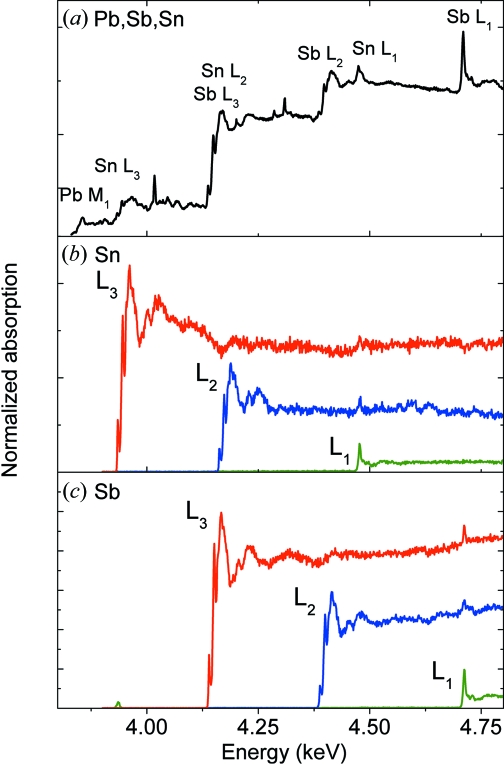
The measured absorption of a Pb–Sb–Sn oxide powder in (*a*) transmission mode, (*b*) fluorescence mode for the Sn *L*α_1,2_ (red), *L*β_1_ (blue) and *L*β_3_ (green) emission lines, and (*c*) fluorescence mode for the Sb *L*α_1,2_ (red), *L*β_1_ (blue) and *L*β_3_ (green) emission lines.

**Figure 9:**
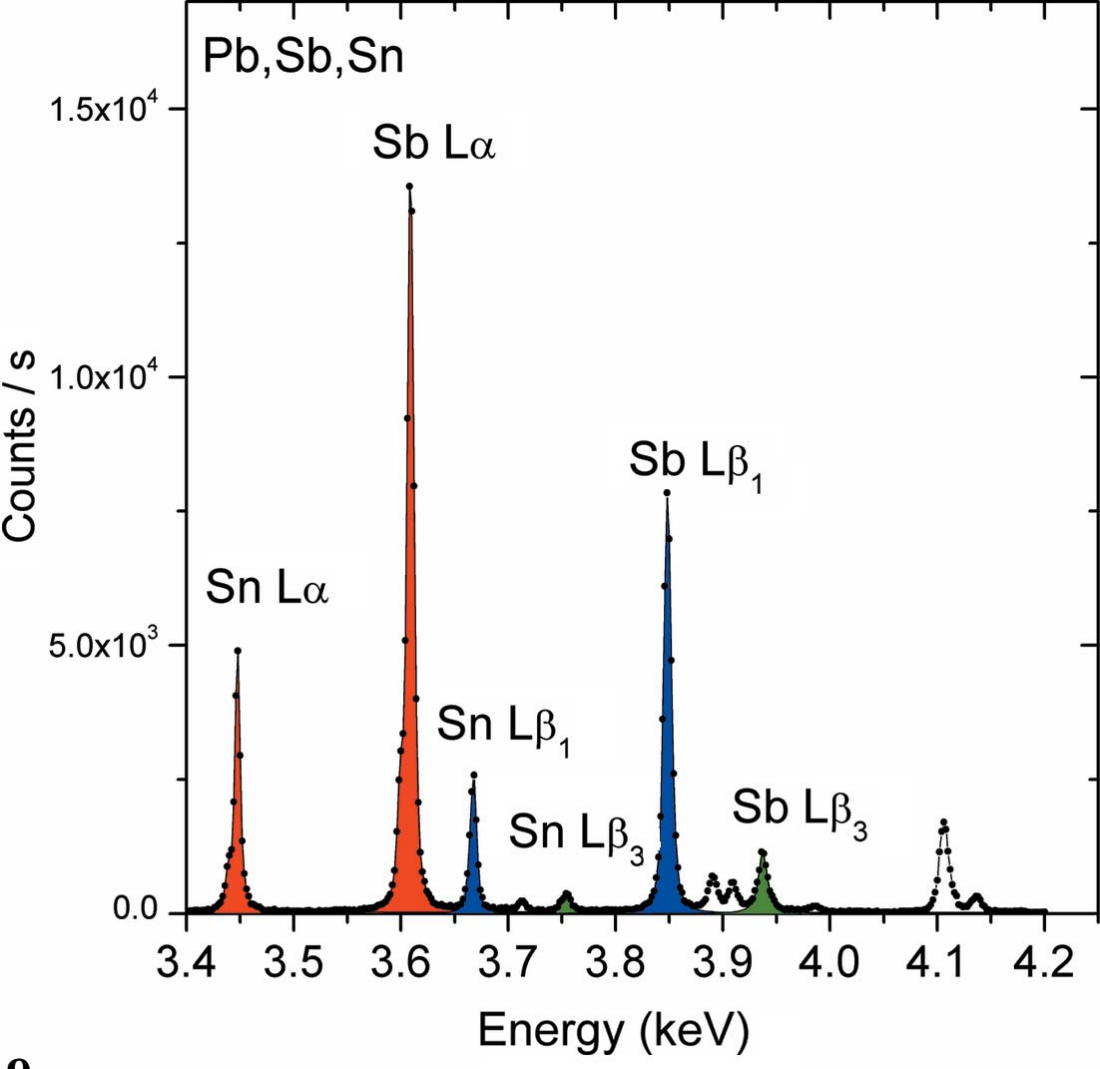
The *L* X-ray fluorescence spectrum from a Pb–Sb–Sn oxide powder. The transitions used for the absorption measurements in the fluorescence mode are marked in colour.

The present example shows the preliminary results of an assessment of the potential of micro-XANES for similar analyses. Owing to the elemental complexity of these compounds, the X-ray fluorescence and XANES analysis is somewhat demanding and difficult. As shown in Fig. 8(*a*) ▶, the absorption spectrum recorded in transmission mode from a reference Pb–Sb–Sn oxide powder (yellow pigment from Kremer) consists of several overlapping *L*- and *M*-absorption edges of Sb, Sn and Pb. The *L*_1_-edge white lines of Sb and Sn are particularly useful as they provide a direct probe of the oxidation state of these elements (Liu *et al.*, 2004 ▶; Dik *et al.*, 2008 ▶). However, the analysis is complicated owing to the large ‘background’ lying below these *L*_1_ edges, and this prevents a proper specification and degrades the detection limits for the Sb and Sn elements. The problem can be solved by recording the XANES spectrum in the fluorescence mode by means of the PB-WDS. As can be seen from the X-ray fluorescence spectrum of Fig. 9 ▶, the resolution of the instrument is more than adequate to separate the main X-ray *L*-fluorescence lines of Sb and Sn. Exploiting the fact that the *L*α_1,2_, *L*β_1_ and *L*β_3_ transitions involve different initial atomic states (*i.e.* *L*_3_, *L*_2_, *L*_1_, respectively), the absorption spectra around the *L*_1,2,3_ edges can be recorded separately by measuring the intensity of the corresponding fluorescence line *versus* beam energy.

A series of absorption spectra were therefore recorded, scanning the beam energy in the 3.9–4.8 keV range with simultaneous acquisition of the fluorescence signals from the sample. For each scan, the PB-WDS was tuned to the Bragg angle corresponding to the *L*α_1,2_, *L*β_1_ or *L*β_3_ fluorescence lines of Sb and Sn. The results of these measurements are shown in Fig. 8(*b*) for Sn and Fig. 8(*c*) ▶ for Sb. As shown, it was possible to separate the XANES spectrum of each element and acquire separately the XANES spectra for the *L*_1_, *L*_2_ and *L*_3_ absorption edges. In particular, the measurement involving the *L*β_3_ emission line allowed for a clear separation and analysis of the *L*_1_ absorption-edge features. The resolution of the X-ray fluorescence detection scheme is of prime importance for such measurements: if a semiconductor detector had been used, the *L*-emission lines would not have been separated and the resulting absorption spectrum, recorded in the fluorescence mode, would have been similar to those presented in Fig. 8(*a*) ▶.

**Figure 10:**
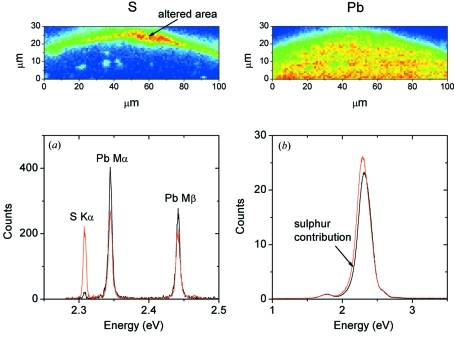
Top panels: X-ray fluorescence maps of S and Pb in the painting cross section containing the altered area. (*a*) The PB-WDS X-ray fluorescence spectrum recorded in the altered (red) and safe (black) areas. (*b*) The same as (*a*) but acquired using a SDD detector.

These preliminary results were obtained on a commercial pigment, conditioned as a powder. We intend to apply this method to historical samples such as ancient glasses and paintings. For such samples, not only spectral resolution will be necessary but also spatial resolution as the pigments and opacifiers are usually present as micrometric particles dispersed in a matrix. Accordingly, a submicrometric probe is crucial to selectively probe the pigments. This point is illustrated in the following analysis example whose objective was to identify the composition of a brown alteration spreading on the surface of a painting named ‘Bateaux de Pêche’, executed by J. A. Noel in 1867 and conserved in the Musée de Picardie, Amiens, France. A sample fragment was taken in an altered area and prepared as a cross section to reveal its entire stratigraphy. The altered brown layer was only 5 µm over the original blue layer, and the main composition of both layers (safe and altered) was lead. The fluorescence spectra measured using a SDD detector showed very small differences between the two layers. In the broad unresolved Pb *M*-lines emission peak, only a shoulder on the low-energy side could be seen (Fig. 10*b* ▶). When acquiring the fluorescence spectra using the PB-WDS, this shoulder can be unequivocally shown to contain the sulfur *K*α emission lines (Fig. 10*a* ▶). The energy resolution of the spectrometer was crucial in this case to establish the presence of sulfur in the brown layer. Spatial micro-mapping of sulfur and lead show that they are both present in the brown layer, while sulfur was absent in the original blue layer (Fig. 10 ▶, top panels). Lead sulfates were subsequently identified using micro-XANES. This analysis showed that the brown alteration observed on the surface of the painting may have resulted from the reaction of the original lead white pigment with exogenous SO_2_.

## 5. Summary

The parallel-beam wavelength-dispersive spectrometer developed at the X-ray microscopy beamline ID21 of ESRF was described. The spectrometer is operated in a flat-crystal geometry employing the polycapillary optics for efficient X-ray fluorescence collection. X-ray-tracing Monte Carlo simulations were performed to determine the main characteristics (transmission, exit divergence) of the polycapillary optics. Experimentally, the detection limits of the spectrometer were found to be at the level of tens of p.p.m. and the energy resolution in the range of tens of eV. This high-energy resolution makes the novel instrument complementary to the solid-state detectors in use at the beamline, opening new possibilities for elemental and chemical analyses combined with submicrometre spatial resolution. It was also shown that further improvement in energy resolution is possible, down to the ∼eV range, by using a double-crystal set-up arranged in an anti-parallel geometry. Finally, examples of X-ray absorption and X-ray fluorescence measurements were presented in which the wavelength-dispersive spectroscopy proved to be essential for a successful elemental and chemical analysis of the samples.

## Figures and Tables

**Table 1:** Polycapillary optics characteristics

Input focal distance	10 mm	Input collection angle	20.04°
Optic output diameter	7.2 mm	Input field of view	50–100 µm
Transmission efficiency at 1.5 keV; at 8 keV	33%; 14%	Output divergence at 8 keV, FWHM	3.4 mrad
